# Book Reviews

**Published:** 1845-06

**Authors:** 


### Bibliographical Notices.

*The Forceps.* Journal of Dental Surgery, and the Collateral Arts and Sciences. London, 1845.

The above is the name of a most excellent and spiritedly conducted journal, published every two weeks. It is in small newspaper form, each No. containing twelve pages. It contains many well written and valuable articles on dental surgery, besides a considerable amount of matter on the collateral arts and sciences. The number before us is 29, of the second volume, but from some cause or other, we have not received more than ten or eleven numbers of the whole series. We should be gratified to receive all the back numbers, and the future ones, regularly, as they are published. It is a paper that should be taken by every dentist desirous of keeping pace with the progress of the science, and we should be glad to have it extensively circulated among the members of the profession in this country. With a view to this, we would suggest to the conductors the propriety of appointing an agent to receive subscriptions in each of the principal cities in the United States. Although not an authorised agent, it would give us pleasure to order it for any one of our professional friends who may be desirous of becoming a subscriber to it. The subscription per annum, in the currency of the United States, is about three dollars and twenty-five cents.—*Balt. Ed.*

*The Dental Mirror, and Brooklyn Annual Visitor, for* 1845. By Martin K. Bridges, D. D. S.

The above is the title of a beautifully gotten up quarto periodical of eighteen pages: designed for the general reader. The subjects treated on, are, generally, well chosen, and presented in such a way, as will, we have no doubt, wherever it circulates, disabuse the public mind of much of the popular error and prejudice under which it has, hitherto, in almost every place, been laboring. Dr. Bridges enjoys the reputation of a scientific and skilful practitioner, and, we trust, his Dental Mirror will, wherever it goes, meet with a kind reception, and exhibit to its numerous readers the importance of a subject which has been, and still is, too much neglected, namely, a sound and healthy dental apparatus. We have always been an advocate for the dissemination of useful knowledge, and, we believe, if the means for the preservation of the health of the teeth, and the cure of their diseases were more thoroughly and generally understood, the people would be less ready, than they are at present, to trust their management to every one advertising himself as a dentist.

In his opposition to every species of dental empiricism, the doctor is unsparing. His remarks, touching certain species of quackery, while they are just, are at the same time severe.

We would be glad to have such a publication circulated throughout every community and neighborhood of the country.—*Balt. Ed.*

